# Supplementary material for: Prenatal Manganese Exposure and Long-Term Neuropsychological Development at 4 Years of Age in a Population-Based Birth Cohort
Source: Int J Environ Res Public Health. 2020 Mar 4;17(5):1665. doi: 10.3390/ijerph17051665 (PMC7084179; doi:10.3390/ijerph17051665)
Supplement: Supplementary file 1 [file ijerph-17-01665-s001.pdf]

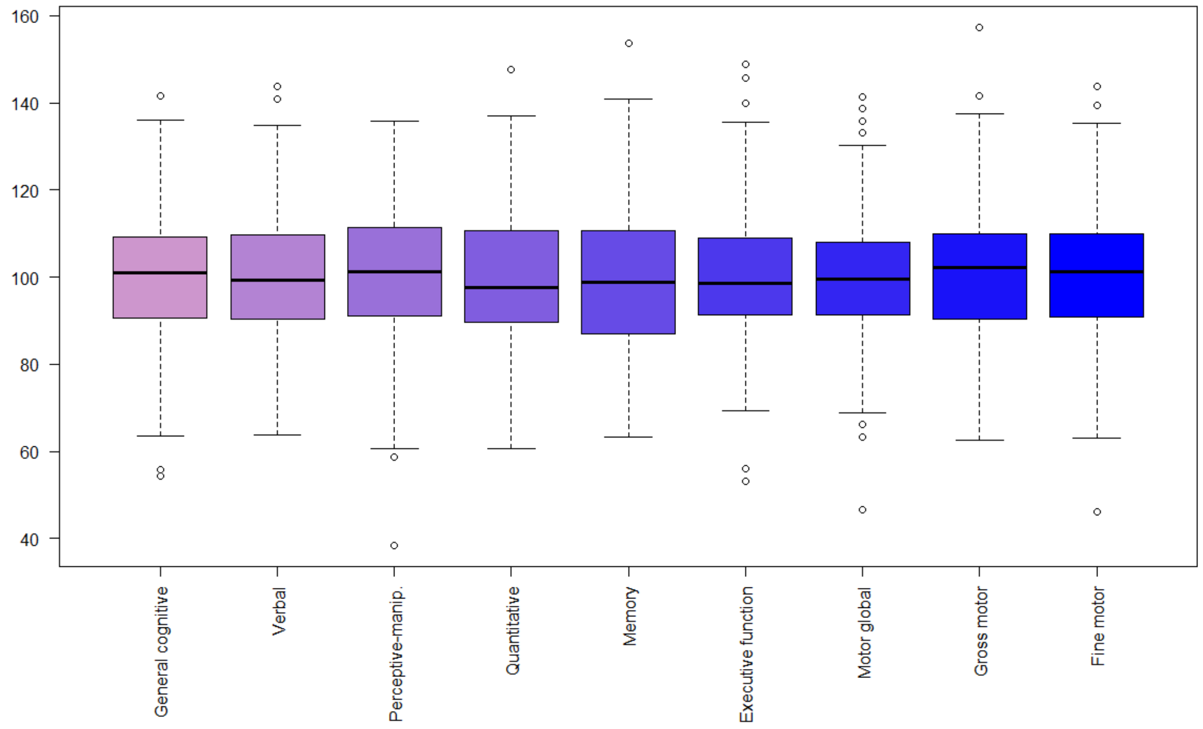

Figure S1. McCarthy scales plots

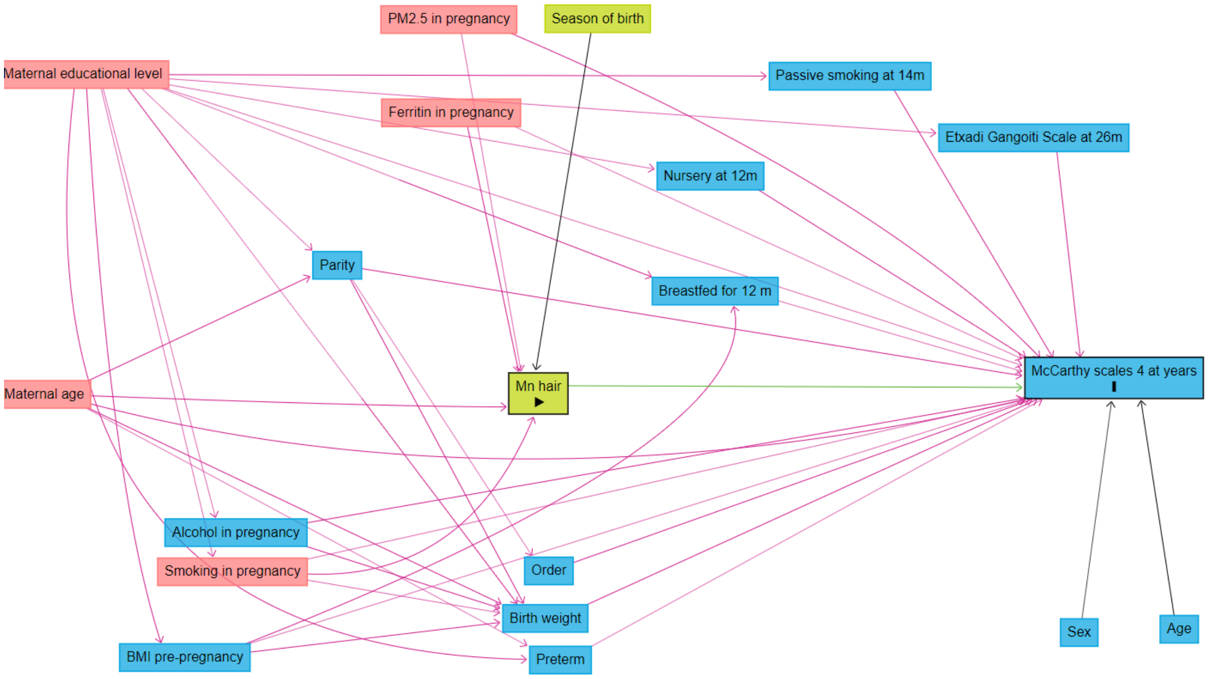

Figure S2. Directed acyclic graph to select the covariates included in the analyses. Note: Yellow node represents the exposure; blue box with an "I" represents the outcome; blue boxes represent the ancestor of the outcome; red boxes represent the confounders. The minimally sufficient

adjustment set included was: $PM_{2.5}$ pregnancy, ferritin in pregnancy, maternal age at conception and smoking in pregnancy.

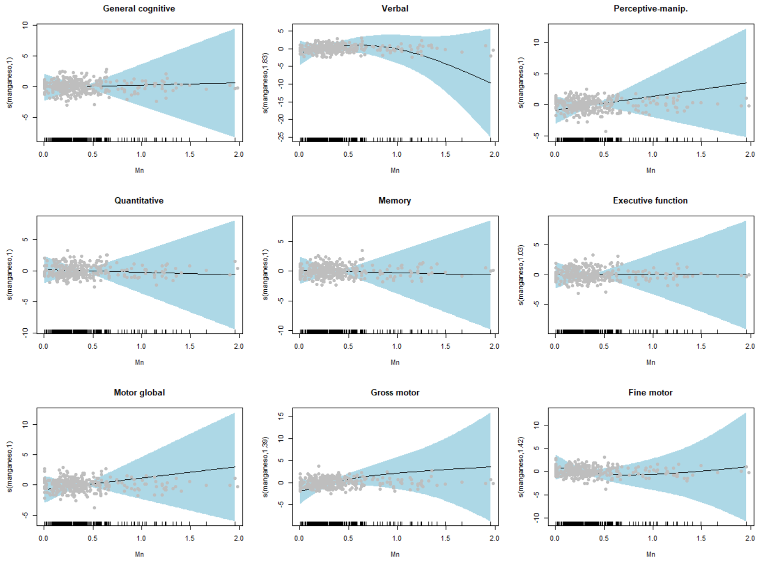

Figure S3. General additive models.

Table S1. Sensitivity analysis: differences between our participants and the whole cohort

		N=304			N=334			
		N (%)	mean (sd)	Number of missing data	N (%)	mean (sd)	Number of missing data	*p*
Mother characteristics								
**Age**			31.67 (3.3)	0(0%)		31.0 (3.95)	0(0%)	***0.029***
**BMI, kg/m²**	<18.5	13 (4.28%)		0(0%)	11 (3.29%)		1 (0.30%)	*0.843*
	18.5≤BMI<25	231 (75.99%)			250 (74.9%)			
	25≤BMI<30	45 (14.8%)			56 (16.8%)			
	BMI≥30	15 (4.93%)			16 (4.79%)			
**Maternal education**	Primary	38 (12.5%)		1 (0.33%)	48 (14.4%)		1 (0.30%)	*0.287*
	Secondary	120 (39.47%)			112 (33.5%)			
	University	145 (47.7%)			173 (51.8%)			
**Parity**	0	167 (54.93%)		0(0%)	178 (53.3%)			*0.758*
	1	116 (38.16%)			136 (40.7%)			
	2+	21 (6.91%)			20 (5.99%)			
**Ferritin level**			34.54 (26.11)	1 (0.33%)		31.3 (28.1)		*0.137*
**$PM_{2.5}$ in pregnancy**			16.98 (2.43)	22 (7.24%)	16.9 (2.49)	83 (24.85%)		*0.726*

Table S1. *Cont.*

**Smoking at week 32**	No	266 (87.5%)		7 (2.3%)	263 (78.7%)		32 (9.58%)	*0.414*
	Yes	31 (10.20%)			39 (11.7%)			

**Alcohol intake during pregnancy**	No	271 (89.1%)	2 (0.66%)	283(95.3%)	37 (11.1%)	*0.272*
	Yes	21 (6.91%)		14 (4.71%)		

T test for continuous variables and chi-squared for categorical variables

Table S2. Linear regression models for the association between Mn and the McCarthy scale scores. Change in the McCarthy scales for 1 μg/g increase in Mn and bytertiles of the Mn distribution including Family Context (Etxadi-Gangoiti SCLD subscale).

			**Beta (CI 95%)**			
**Scale**		**Tertile 1 [0.0065 - 0.2316]**	**Tertile 2 (0.2316- 0.4093]**	**Tertile 3 (0.4093 - 2.1658]**	***p for trend***	**By 1 point increment in the Mn**
**General cognitive**	**Model 1**	1 (Ref.)	0.75 (-3.79, 5.3)	-0.68 (-5.37, 4.02)	*0.784*	-1.34 (-7.06, 4.37)
**Verbal**	**Model 1**	1 (Ref.)	1.08 (-3.77, 5.92)	0.03 (-4.96, 5.02)	*0.983*	-2.11 (-8.19, 3.97)
**Perceptive-manip.**	**Model 1**	1 (Ref.)	0.07 (-4.47, 4.61)	-0.43 (-5.12, 4.25)	*0.857*	0.72 (-4.99, 6.43)
**Quantitative**	**Model 1**	1 (Ref.)	0.2 (-4.44, 4.84)	-1.67 (-6.46, 3.12)	*0.497*	-2.48 (-8.31, 3.36)
**Memory**	**Model 1**	1 (Ref.)	1.3 (-3.54, 6.14)	0.79 (-4.2, 5.79)	*0.749*	-1.62 (-7.7, 4.47)
**Executive**	**Model 1**	1 (Ref.)	1.01 (-3.56, 5.58)	-0.69 (-5.4, 4.03)	*0.783*	-1.82 (-7.57, 3.92)
**Motor global**	**Model 1**	1 (Ref.)	0.64 (-3.99, 5.28)	-0.8 (-5.59, 3.98)	*0.747*	0.01 (-5.82, 5.85)
**Gross motor**	**Model 1**	1 (Ref.)	3.47 (-1.3, 8.24)	1.38 (-3.54, 6.3)	*0.566*	1.85 (-4.17, 7.87)
**Fine motor**	**Model 1**	1 (Ref.)	-2.75 (-7.11, 1.6)	-2.7 (-7.2, 1.79)	*0.231*	-1.98 (-7.47, 3.52)

Model 2**: also adjusted for age at the time of the test, sex, sibling order, nursery at 12 months and maternal education and Etxadi-Gangoiti Scale (Stimulation of Cognitive and Linguistic Development subscale).
